# Optimisation of Shrinkage and Strength on Thick Plate Part Using Recycled LDPE Materials

**DOI:** 10.3390/ma14071795

**Published:** 2021-04-05

**Authors:** Norshahira Roslan, Shayfull Zamree Abd Rahim, Abdellah El-hadj Abdellah, Mohd Mustafa Al Bakri Abdullah, Katarzyna Błoch, Paweł Pietrusiewicz, Marcin Nabiałek, Janusz Szmidla, Dariusz Kwiatkowski, Joel Oliveira Correia Vasco, Mohd Nasir Mat Saad, Mohd Fathullah Ghazali

**Affiliations:** 1 Faculty of Mechanical Engineering Technology, Pauh Putra Main Campus, Universiti Malaysia Perlis, Perlis 02600, Malaysia; shahiraroslan@yahoo.com (N.R.); nasirsaad@unimap.edu.my (M.N.M.S.); fathullah@unimap.edu.my (M.F.G.); 2 Center of Excellence Geopolymer and Green Technology (CEGeoGTech), Universiti Malaysia Perlis, Perlis 01000, Malaysia; mustafa_albakri@unimap.edu.my; 3 Laboratory of Mechanics, Physics and Mathematical Modelling (LMP2M), University of Medea, Medea 26000, Algeria; lmp2m_cum@yahoo.fr; 4 Faculty of Chemical Engineering Technology, Pauh Putra Main Campus, Universiti Malaysia Perlis, Perlis 02600, Malaysia; 5 Department of Physics, Faculty of Processing Engineering and Materials Technology, Częstochowa University of Technology, 42-200 Częstochowa, Poland; katarzyna.bloch@pcz.pl (K.B.); pawel.pietrusiewicz@pcz.pl (P.P.); nmarcell@wp.pl (M.N.); 6 Faculty of Mechanical Engineering and Computer Science, Częstochowa University of Technology, 42-200 Częstochowa, Poland; j.szmidla@imipkm.pcz.pl (J.S.); kwiatkowski@ipp.pcz.pl (D.K.); 7 Department of Mechanical Engineering, School of Technology and Management (ESTG), Polytechnic of Leiria, 2411-901 Leiria, Portugal; joel.vasco@ipleiria.pt

**Keywords:** response surface methodology, particle swarm optimisation, recycled low-density polyethylene (LDPE), shrinkage, strength

## Abstract

Achieving good quality of products from plastic injection moulding processes is very challenging, since the process comprises many affecting parameters. Common defects such as warpage are hard to avoid, and the defective parts will eventually go to waste, leading to unnecessary costs to the manufacturer. The use of recycled material from postindustrial waste has been studied by a few researchers. However, the application of an optimisation method by which to optimise processing parameters to mould parts using recycled materials remains lacking. In this study, Response Surface Methodology (RSM) and Particle Swarm Optimisation (PSO) methods were conducted on thick plate parts moulded using virgin and recycled low-density polyethylene (LDPE) materials (100:0, 70:30, 60:40 and 50:50; virgin to recycle material ratios) to find the optimal input parameters for each of the material ratios. Shrinkage in the *x* and *y* directions increased in correlation with the recycled ratio, compared to virgin material. Meanwhile, the tensile strength of the thick plate part continued to decrease when the recycled ratio increased. R30 (70:30) had the optimum shrinkage in the *x* direction with respect to R0 (100:0) material where the shrinkage increased by 24.49% (RSM) and 33.20% (PSO). On the other hand, the shrinkage in the *y* direction for R30 material increased by 4.48% (RSM) and decreased by 2.67% (PSO), while the tensile strength of R30 (70:30) material decreased by 0.51% (RSM) and 2.68% (PSO) as compared to R0 (100:0) material. Validation tests indicated that the optimal setting of processing parameter suggested by PSO and RSM for R0 (100:0), R30 (70:30), R40 (60:40) and R50 (50:50) was less than 10%.

## 1. Introduction

Plastic injection moulding is a well-known process for high-volume production of plastic parts, such as automotive, medical and electronic products, to meet the market demand. The quality of moulded parts does not depend solely on one factor; it is affected by several factors such as part design, input parameters, selection of material and mould design [1]. Therefore, the ability to produce a part of good quality is of the utmost importance. Finding the optimal setting of the processing parameter is the most vital step to improving the quality of the moulded parts [2].

The use of recycled materials affects the part properties, especially strength. The use of trial-and-error is one of the options available to optimize the associated parameters, but this approach is not suitable for current industries using complex manufacturing processes, as it is time-consuming and costly [3]. Therefore, applying optimisation methods such as the Response Surface Methodology (RSM), Genetic Algorithm (GA), Particle Swarm Optimisation (PSO) and Taguchi is a good option to optimise the setting of processing parameters to mould parts with better quality [4,5,6,7,8,9,10,11,12].

RSM is among the optimisation methods that have been used by many researchers [6,7,8,9,10]. Sudsawat and Sriseubsai [10] conducted research using the RSM optimisation approach to create optimal process settings to minimise warpage and volume shrinkage. General Purpose Polystyrene (GPPS) was used as the material. Eighty-four trials of central composite design (CCD) were developed and data simulation was performed using simulation tools. The Firefly Algorithm (FA) and Genetic Algorithm (GA) were used to find the optimal minimum shrinkage and warpage value of the part. The results that were acquired through these methods were compared with the experimental work. Seven parameters were studied, namely, the mould temperature, packing pressure, cooling time, melt temperature, packing time, packing pressure and flow rate profile. The analysis results showed that melt temperature and cooling time were most affected shrinkage and warpage. Besides that, FA was found to yield better shrinkage and warpage results than the GA, with differences of 4.05% (simulation) and 3.28% (experimental), compared to 7.4% (simulation) and 10.26% (experimental), respectively, with GA.

Chen et al. [6] used Taguchi and RSM methods to establish input parameters for the warp of plastic spur gears produced using polyoxymethylene (POM) content. The input parameters analysed were packing pressure, holding pressure, melt temperature, holding time and mould temperature. The optimal minimum warpage produced by Taguchi was compared with RSM. The Taguchi method showed that holding time was the most influential factor on part warpage. By using the best set of parameters, the error recorded between the experimental work and simulation study was only 2.07%.

Azman et al. [11] applied the PSO to optimise the injection mould parameters by obtaining the minimum warpage, whereby the parameters studied were cooling temperature, V/P switchover, injection time and mould temperature. The regression model that represents the relationship between the response and processing parameters was acquired from the previous researches [12]. As compared to the warpage value from the previous study, the warpage recorded a reduction of 2.21%, proving the ability of PSO to optimise the warpage problem.

Variables such as the materials, processing parameter and design were seen to have an impact on the finished moulded part [13]. Optimisation methods such as the Taguchi process and RSM have been used over the years and have shown exceptional performance in the production of better quality of moulded parts [6,7,12,14,15]. However, only a few researchers have applied these optimisation methods to reduce shrinkage and improvise the tensile properties of a recycled material [16,17]. Most researchers have used the optimisation technique solely to test the tensile properties of recycled plastics [16,17,18,19,20].

Fei et al. [16] studied the input parameters on parts moulded using recycled ABS materials. The Taguchi method was employed, whereby four input parameters were studied. The parameters were melt temperature, packing pressure, packing time and injection time. The output observed comprised shrinkage, elongation at breaking point and tensile strength. Recycled material that was used for manufacturing these parts was reported to be better than virgin material when the optimum input parameters were employed. Additionally, it was seen that the hoop tensile strength, elongation at break and shrinkage improved by 2.33%, 23.52% and 93.3% respectively, after optimisation.

Abdullaha et al. [17] studied the optimal setting of processing parameters by using the Taguchi method on a plastic tray made of recycled high-density polyethylene (HDPE). The quality characteristics investigated were shrinkage, tensile strength and flexural strength. It was reported that the most significant processing parameter affecting the shrinkage and flexural strength was packing time, while melting temperature was reported to be the most significant processing parameter affecting tensile strength.

Similarly, Meran et al. [21] reported the potential use of recycled low-density polyethylene (LDPE), HDPE and polypropylene (PP) to improve tensile strength. Sets of virgin and recycled materials were mixed in a number of different compositions, and later, their tensile strength was examined. The results showed that tensile strength was reduced by 15% when the virgin LDPE content was reduced. The tensile strength was reduced to 5% when the proportion of new HDPE was reduced. It was also claimed that the tensile strength decreased to 3% when the proportions of new HDPE and LDPE in the content mixture were decreased.

Javierre et al. [22] investigated the impact of the recycled content mixed with virgin material, whereby mechanical properties and the safety factor of the moulded component were set as the outputs. Recycled content was mixed into different percentages; the findings showed that stress at yield for 100% recycled HDPE material decreased by 20% compared to the fully virgin material. Meanwhile, the material blended with 40% recycled material had a minimal effect on safety factor as compared to the stress module. The tensile modulus of 100% recycled HDPE material was reduced by 40% compared to that of virgin HDPE. Additionally, the effect of mixing recycled material below 60% was found to be less significant on the tensile modulus (which was only 15%).

Literature reviews have indicated that the optimisation of injection moulding parameters can be employed to achieve the desired performance [17,23]. So far, this optimisation method has only been applied by a few researchers to increase part quality (in terms of tensile properties) using recycled plastic material [24,25]. Meanwhile, most research has only focused on changes in material properties due to variations in the ratio of recycled materials mixed with the virgin materials [18,19,20,21,22,25,26,27,28,29]. Therefore, it is necessary to conduct deep research in order to determine the full potential of reusing recycled material, especially post-industrial waste, which is easy to control from mixing with other types of materials. In this way, manufacturers can fully utilise raw materials in injection moulding processes.

LDPE is a polyolefins material which is widely used in injection moulding process [30]. It is used to mould many types of products such as laboratory apparatuses and household products. Compared to other popularly used polyolefins materials in past research, studies conducted using LDPE are rather few in number. Since few works have examined this material, it would be of huge advantage to study its performance when it is recycled.

In this study, rejected thick plate parts, including the sprue, runner and gating of LDPE materials, were recycled. The recycled materials were divided into virgin:recycled ratios of 70:30 (R30), 60:40 (R40) and 50:50 (R50). Optimisation methods (RSM and PSO) were applied to optimise the shrinkage and strength of the thick plate part of virgin (R0), R30, R40 and R50 materials in simulation studies [31] using Autodesk Moldflow Insight (AMI) 2012, and were validated with experimental works. This study provides guidance to moulding industries on the effect of simulations and experimental works with integrated optimisation methods and the effect of the recycle ratio on shrinkage and on the strength on the moulded parts. The findings benefit mould industries seeking to use recycled materials while reducing the manufacturing cost without compromising on quality. The long-term implication of this study is its contribution to the global effort to sustain the environment.

## 2. Methods and Materials

**Figure 1:**
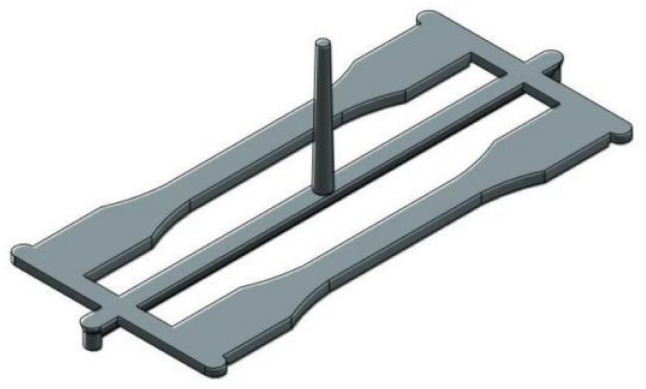
Thick plate part.

**Figure 2:**
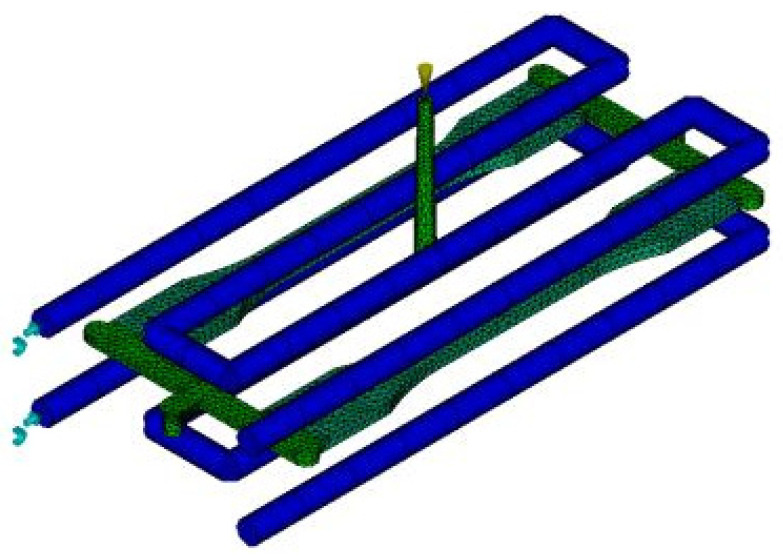
Thick plate part with cooling system.

The thick plate part was designed as depicted in Figure 1. The meshes, feed system and cooling system are shown in Figure 2. The thick plate part chosen for this study has a length of 150 mm, the width of 20 mm and thickness of 4 mm moulded using LDPE material. A 3D solid mesh in a tetrahedral form with four nodes covers the whole part and gating system, as shown in Figure 1. The meshed part and gating system consist of 345,914 tetrahedral elements, connected by 64,123 nodes. The ratio for the volume feed system to the volume of the part is 0.56, which is volumetrically adequate. Meanwhile, as shown in Figure 2, a cooling system was developed with a 6 mm diameter of the beam elements, as suggested by AMI software (Autodesk Moldflow Insight 2012, Moldflow, Melbourne, Australia). Eighty-eight beam elements were used to form the cooling system with a total of 90 nodes.

Autodesk Moldflow provides information on material properties for more than 10,000 types of plastic [32]. The details of the material properties used are listed in Table 1. LDPE, with the commercial name Sumikathene 206P, manufactured by Prime Polymers Co. Ltd. (Tokyo, Japan), was selected from the AMI database for use in simulations study; the same grade was used in the experimental study. The database from AMI 2012 was available for only the virgin material, while there were no data available for the specific virgin to recycled ratios used in this study. The virgin to recycled ratio materials used in this study were 70:30, 60:40 and 50:50 respectively. The ratio of materials was denoted as R0 for virgin specimens, R30 for 70:30, R40 for 60:40 and R50 for 50:50 on virgin to material ratio specimens.

### 2.1. Response Surface Methodology (RSM)

**Figure 3:**
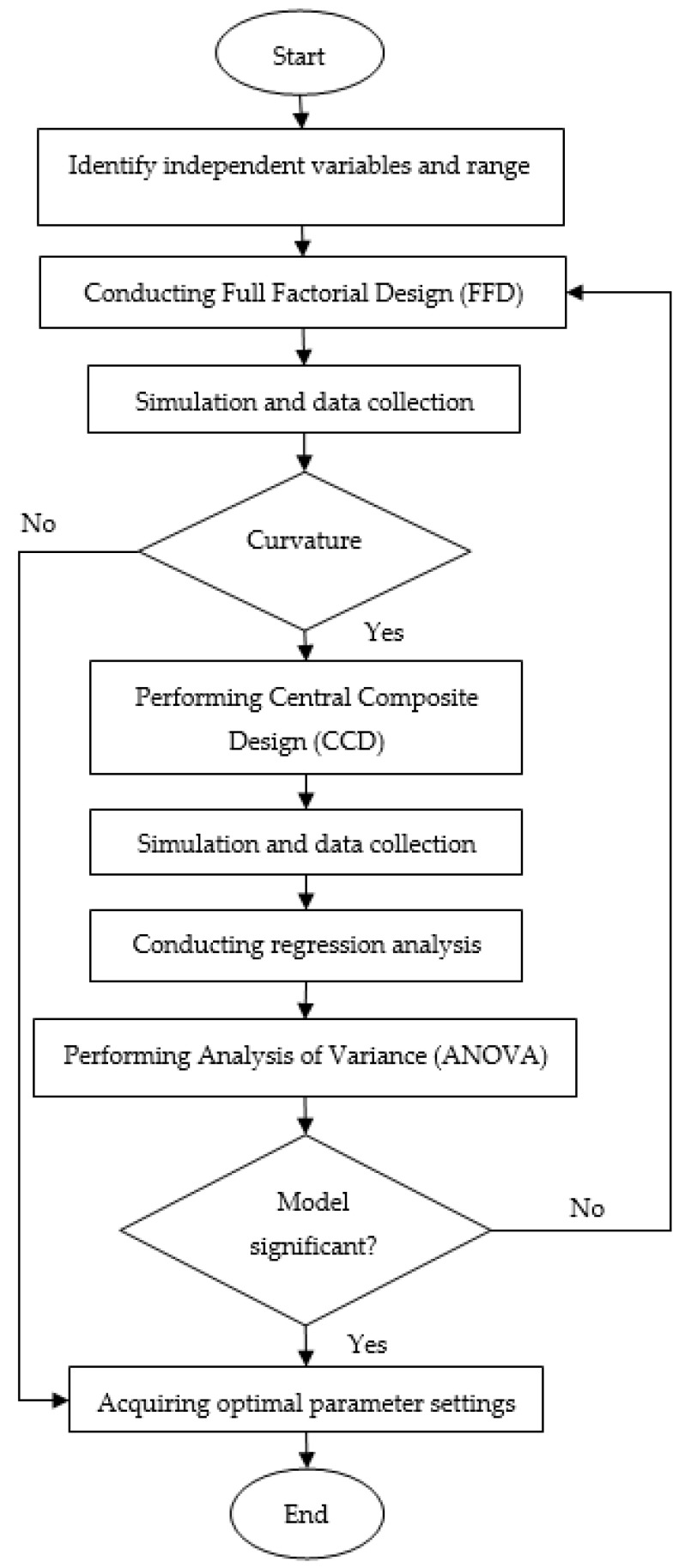
The RSM’s flowchart.

RSM was chosen as it can determine the relationships between several input variables relative to response variables. This statistical method is widely applied along with other optimisation methods to solve either single or multiple objective problems in various research areas [34]. Figure 3 illustrates the process flow of the RSM. RSM provides data to evaluate the effect of each input variable regarding shrinkage and strength in order to determine the optimal settings of the processing parameters.

#### 2.1.1. Full Factorial Design

Mould temperature, melt temperature, packing pressure and cooling time were selected as the input parameters due to their influence on the shrinkage and tensile strength, as stated in the literature [13,17,32,35,36,37]. An AMI 2012 simulation software was used to determine the input parameters, as shown in Table 2. The software can also determine the maximum and minimum range of the input parameters, as seen in Table 3. Packing pressure and cooling time min and max range were determined based on the Fill+Pack analysis and cool (FEM) analysis in the AMI software. The maximum value for packing pressure and cooling time (output) were determined when all of other processing settings (input) in Table 3 were set to the maximum values. Meanwhile, the minimum value for packing pressure and cooling time (output) were determined when all other parameter settings (input) in Table 3 were set to the minimum values. Table 1 shows the mould and melt temperatures obtained from the material properties. The mould temperature was in the range between 20–60 °C, as recommended by the supplier. It was also recommended that melt temperatures remain between 160–180 °C.

For design of experiments (DOE) analysis, Design Expert software was employed to assist the computation of the full factorial. In the full factorial, a set of 20 combination settings of processing parameters with four centre points was formed. The set of processing parameters was formed based on the range of processing parameters shown in Table 3 as the input parameter. The ANOVA in FFD evaluates the model as well as eliminating the insignificant input parameters that have probability values more than 0.05 (*p*-value > 0.05) and are considered to influence the shrinkage and tensile strength of the thick plate part. The presence of curvature in the ANOVA shows that the quadratic model is a more suitable fit in comparison to the linear model [38]. Hence, augmentation of FFD adds another 10 settings of processing parameters into the prior 20 settings of processing parameters. This is called the face centred central composite design (CCD), with a total of 30 combinations of parameters, as tabulated in Table 4. The additional 10 settings of processing parameters are to add points to the quadratic shaped graph and provide a better representation of the interaction between parameters and responses.

#### 2.1.2. Face Centred Central Composite Design (CCD)

As previously mentioned, Face Central Composite Design (CCD) was employed to search for the correct fitting with the quadratic model of RSM. As shown in Table 4, in conducting the CCD, ten extra settings for the input parameters were added to the list. Again, a collection of data for the ten extra settings of input parameters was conducted through simulation of AMI and the injection moulding machine (Nissei NEX1000, Nissei Plastic Industrial Co., LTD., Minamijo, Japan). Through the regression analysis, a mathematical model showing the correlation between the output responses and input parameters was then generated. The mathematical models representing each of the responses was further used to conduct PSO optimization [34,39,40].

### 2.2. Particle Swarm Optimisation

**Figure 4:**
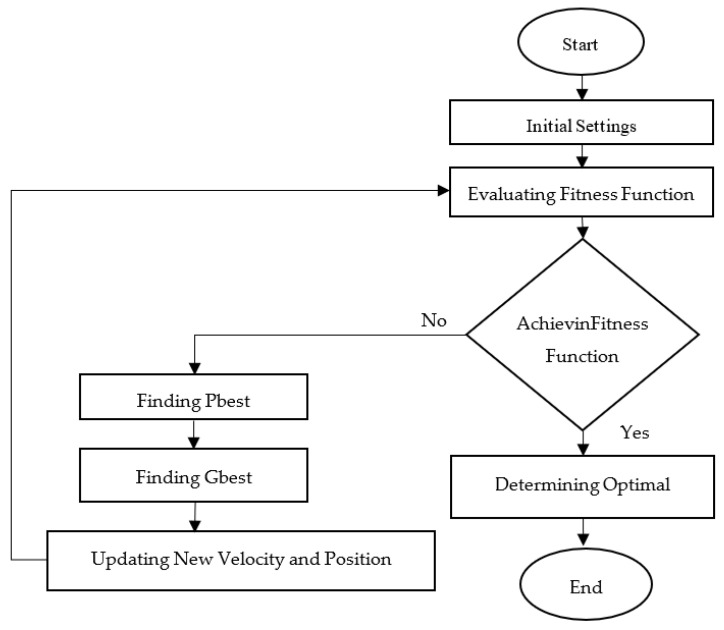
PSO flowchart.

In 1995, Professor Eberhant and Dr Kennedy developed the PSO method, which was inspired by the social behaviour of flocking birds. Figure 4 shows a flowchart of PSO. The initial settings were made whereby the maximum and minimum range of the input parameters were set [41]. Additionally, the numbers of particle iterations and populations were also defined. Each time the programme evaluated a new solutions, the solution was calculated through the fitness function. The objective function gained from the RSM mathematical model was used as the fitness function to select the best solutions to produce in each iteration. In each iteration, the best solution of each particle was evaluated and recorded as $P_{i}=\left(P_{i1},P_{i2},\ldots P_{id}\right)$, also known as the Pbest. Next, the best solution produced by particles in the population were selected and recorded as $P_{g}=\left(P_{g1},P_{g2},\ldots P_{gd}\right)$, known as the gbest. The new velocity and position of each particle were updated for the particle movement in the next iteration. The velocity and position were updated by applying Equations (1) and (2).

The new gbest may be compared with the previous one, and the gbest with the best solution will be selected as the new gbest, before the iteration continues. The stopping criterion is when the predefined number of iteration sets has been completed or the same solution is produced in multiple iterations (i.e., the optimal solution has been achieved).

(1) $$v_{ij}{}^{k+1}=\omega v_{ij}{}^{k}+c_{1}r_{1}\left[P_{ij}-x_{ij}{}^{k}\right]+c_{2}r_{2}\left[P_{gj}-x_{ij}{}^{k}\right]$$

(2) $$x_{ij}{}^{k+1}=x_{ij}{}^{k}+v_{ij}{}^{k+1}$$

### 2.3. Experimental Work

The AMI 2012 and injection moulding machine were both used for collecting the data for R0 material, as seen in Table 4. For accurate results, the injection moulding machine specifications (Nissei NEX 80 tonnes) and the mould material specification (P20 steel) were set in the AMI 2012 software. The simulation of shrinkage study was conducted through the Cool (FEM)+Fill+Pack+Warp analysis. Meanwhile, in the experimental work, the shrinkage was manually measured using Digital calliper, while the tensile strength was evaluated using the Universal Testing Machine.

**Figure 5:**
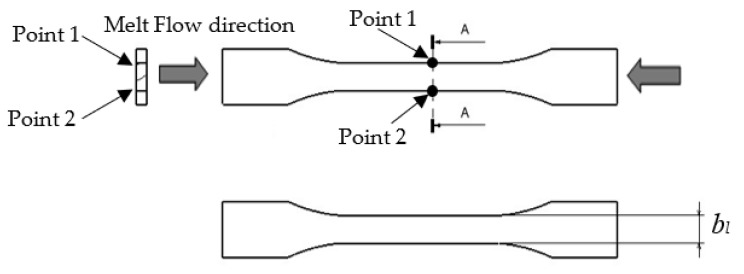
Point measurement in the *x* direction.

**Figure 6:**
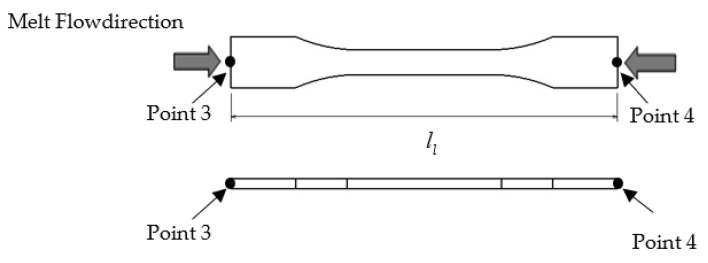
Point measurement in the *y*-direction.

Using the same list for the setting of processing parameters in Table 4, the data of the thick plate part moulded using R30, R40 and R50 specimens were collected. The experimental tests on shrinkage of the thick plate part in the *x* and *y* directions and tensile strength using R0, R30, R40 and R50 specimens were conducted using the injection moulding machine, UTM machine (tensile test) and digital caliper (shrinkage measurement). The performance of the R30, R40 and R50 specimens were compared with that of the R0 specimen. The tests on shrinkage of the thick plate part in the *x* and *y* directions were calculated using Equations (3) and (4), at the point shown in Figure 5 and Figure 6, respectively. Information on the tensile strength of the thick plate part was acquired by using Universal Testing Machine (UTM, Shimadzu Corporation, Kyoto, Japan) with a testing speed of 50 mm/min based on ISO 527 Part 1 [42]. The shrinkage of the thick plate part in the *x* and *y* directions was measured using a digital calliper with an accuracy of 0.01 mm based on ISO 294-1 standard [43]. The measurement of the thick plate part was conducted 48 h after the parts had been injected out [17].

(3) $$S_{x}=100\;\frac{b_{c}-b_{l}}{b_{c}}$$

(4) $$S_{y}=100\;\frac{l_{c}-l_{l}}{l_{c}}$$

## 3. Results and Discussion

### 3.1. Results of Simulation Studies

Through the FFD, the contribution of each input parameter on *x* and *y* directions of shrinkage are shown in Table 5. It can be observed that the most significant processing parameter affecting the shrinkage in both directions was the packing pressure. This is due to the fact that in order to compensate the part volume reduction due to cooling, the right packing pressure allows enough molten plastic to be injected into the remaining mould cavity, hence reducing the shrinkage on the moulded part. Meanwhile, the least significant processing parameter on shrinkage in the *x* and *y* directions as shown in the table was the cooling time.

Through the ANOVA in CCD, the mathematical model was then developed to examine the relationship between processing parameter and responses. The mathematical model was formed in second-order polynomials equations, as shown in Equations (5) and (6) and was then used to conduct the optimisation of PSO. PSO generate processing parameters values and the polynomials equations formed in CCD were used to calculate the shrinkage values. The results from RSM and PSO methods are shown in Table 6. As illustrated in the table, the part moulded using the recommended setting of processing parameters, RSM and PSO showed better shrinkage percentages. The shrinkage in the *x* direction of RSM and PSO improved 8.71% as compared to the part moulded using the recommended setting. Meanwhile, shrinkage in the *y* direction for RSM and PSO improved 8.64% and 9.73% respectively as compared to the part moulded using the recommended setting.

*x* = 5.78555 − 0.053559A − 0.015490B − 0.12845C − 0.03688D + 0.00025AB + 0.0003931AC + 0.00035AD + 0.0003931BC + 0.001104CD (5)

*y* = −3.6694 − 0.041347A + 0.069879B + 0.027560C + 0.015196D + 0.000213AB + 0.000369AD + 0.000295BC − 0.00021BD − 0.00023B^2^ − 0.00298C^2^ (6)

### 3.2. Results of Simulation Study and Experimental Work Using Virgin Material

**Figure 7:**
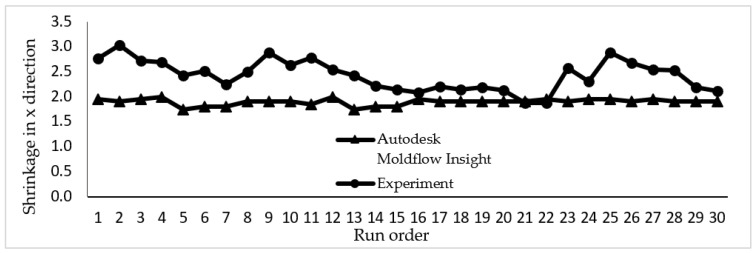
The shrinkage percentages in the *x* direction conducted in simulation study and experimental work.

**Figure 8:**
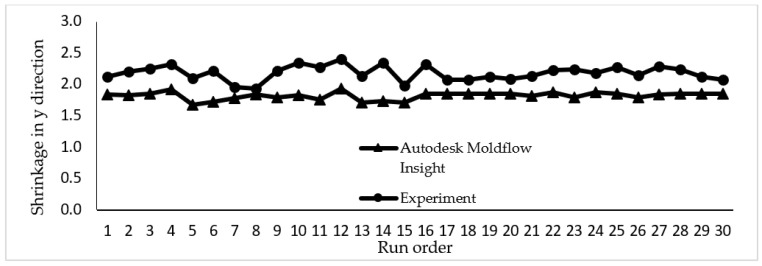
The shrinkage percentage in *y*-direction conducted in simulation study and experimental work.

The data collected from a simulation study and experimental work for shrinkage in the *x* and *y* directions are plotted in Figure 7 and Figure 8. Both graphs showed that shrinkage percentage in the *x* and *y* directions are in a good agreement for both the simulation and experimental works. There is a slight variation as seen in the graph due to several parameters in the simulation such as mould temperature that are assumed to be constant throughout the simulation process.

### 3.3. Experimental Works Results

Through the FFD, the contributions of each input parameters are tabulated in Table 7. The table indicates that packing pressure is the most significant processing parameter that affects the shrinkage in the *x*-direction. The results are in line with those of Liao et al. [44], where packing pressure is the most critical processing parameter that affects the shrinkage of part. The packing pressure pushes molten plastic into the mould as the moulded parts are cooled and shrank inside the mould cavities. The change in packing pressure regulates the compression of the melt along the flow path. Excessive shrinkage can happen if the packing pressure applied is insufficient. Meanwhile, mould temperature is seen to be the most significant processing parameter affecting shrinkage in *y*-direction for thick plate part moulded using recycled material (R30, R40 and R50). A high mould temperature allows more time for crystallisation to occur. An increase in crystallisation produces a high-density part, causing less shrinkage formation. This condition shows that changes in mould temperature and packing pressure result in an increase or decrease of shrinkage percentages in the *x* and *y* directions. Besides, it can be seen that the packing pressure was the most influential parameter affecting the tensile strength for parts moulded using R0 and R30 specimens. Increasing the packing pressure will increase the crystallinity of the materials as the polymer’s crystallinity increases in strength, the intermolecular bonding becomes more significant in the crystalline phase [45,46,47]. Meanwhile, mould temperature is the most significant parameter affecting the tensile strength for parts moulded using R40 and R50. It was seen from the past works that an increase in mould temperature increases the material crystallinity thus producing parts with good tensile strength [32]. This is because the rise in mould temperature increases the plastic density and tensile strength of the moulded parts produced.

**Figure 9:**
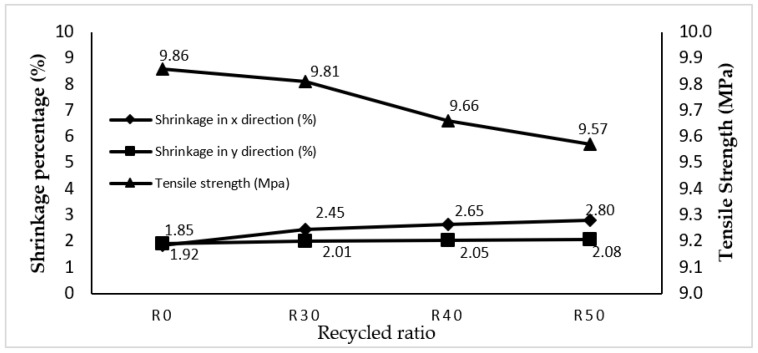
RSM optimisation method on the thick plate part for shrinkage and tensile strength.

**Figure 10:**
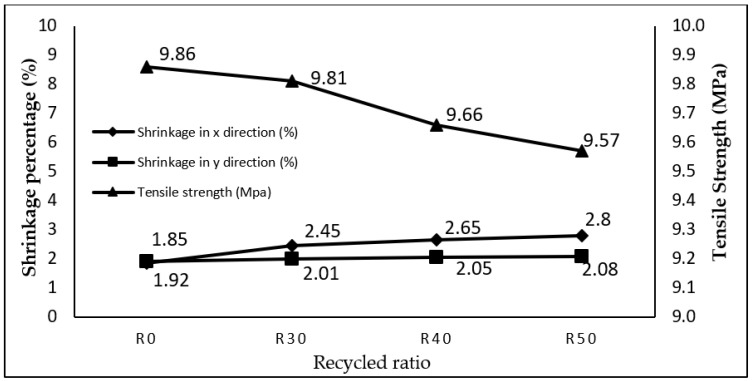
PSO optimisation method on the thick plate part for shrinkage and tensile strength.

Through the CCD, a mathematical model denoting the interaction between the response and input parameters was determined. The shrinkage of the thick plate part in the *x* and *y* directions and the tensile strength were optimised to the values as shown in Table 8 using the RSM optimisation method, and the results are plotted in Figure 9. In addition, Table 8 shows the optimal shrinkage of thick plate part in the *x* and *y* directions and tensile strength for thick plate part optimised using the PSO method. Subsequently, these results are plotted in Figure 10.

Compared with the results of R0 (RSM) shown in Table 8, it is apparent that the result of using RSM on R30, R40 and R50 in the *x* direction increased by 24.49%, 30.19% and 33.93% respectively, while shrinkage in the *x* direction, optimised using the PSO method, increased by 33.20%, 37.82% and 35.95% compared to R0 (PSO). Meanwhile, shrinkage improvement via the use of RSM for R30, R40 and R50 specimens improved by 4.48%, 6.34% and 7.69% respectively in the *y* direction, as compared to R0 (RSM). Shrinkage in the *y* direction optimised using PSO method for R40 and R50 increased by 4.48% and 35.95% respectively, while R30 decreased by 2.67% compared to R0 (PSO). Lastly, tensile strength optimised by RSM for R30, R40 and R50 specimens decreased by 0.51%, 2.07% and 3.03% respectively, as compared to R0 (RSM), while tensile strength for R30, R40 and R50 specimens was decreased using PSO method by 2.68%, 2.68% and 3.64% respectively. What is interesting in Figure 9 and Figure 10 is that the closest recycled material ratio performance to the virgin is produced using R30 specimen. On top of that, the optimal shrinkage and tensile value generated by RSM and PSO did not exceed the maximum tensile strength value and maximum shrinkage rate. Table 8 also shows the optimal processing parameters produced by both methods which are applicable to mould the thick plate part.

### 3.4. Validation Test Results

Table 9, Table 10 and Table 11 present the results of the validation test for shrinkage in the *x* and *y* directions and tensile strength of the specimens by applying the optimal settings of processing parameters as suggested from the RSM and PSO analysis for R0, R30, R40 and R50 specimens. The validation data is quite revealing in such a way that the percentage differences between the results achieved from the validation test in comparison with the optimisation method has minimal error of between 0.21% to 8.07%, indicating that optimal settings of processing parameters produced by optimisation methods were acceptable to mould parts made of recycled LDPE, since the percentage errors are all below 10% [17,48].

## 4. Conclusions

Using a combination of virgin and recycled materials has a great impact on the quality of thick plate parts moulded through the injection moulding process. The shrinkage and strength were optimised by using the RSM and PSO methods. Several conclusions can be drawn from this study:

- Based on the ANOVA analysis conducted in FFD, the most significant parameter influencing the shrinkage in the *x* direction is the packing pressure for all virgin to recycled ratio materials. Meanwhile, recycled materials of R30, R40 and R50 showed that the mould temperature is the most significant parameter influencing the *y* direction of shrinkage. Lastly, packing pressure was found to be the most significant processing parameter affecting the strength of R0 and R30, while mould temperature is the most significant processing parameter affecting the strength of R40 and R50.
- The shrinkage in the *x* and *y* directions continues to increase when the amount of recycled ratio increases as compared to fully virgin material, while the tensile strength of the thick plate part continues to decrease when the amount of recycled ratio increases.
- After applying the RSM and PSO methods, the specimen with 30% recycled material (R30) showed the closest shrinkage and tensile strength quality performance to the R0 specimen.
- The validation test results indicated that the optimal setting of processing parameters, as suggested by PSO and RSM optimisation methods, is acceptable, since the errors were all below 10% as compared to the real measured data.

## Figures and Tables

**Table 1:** Materials Properties of Low Density Polyethylene (LDPE) (Reproduced from [33], with the permission of AIP publishing).

Plastic Material	LDPE
Trade name/Grade	Sumikathe 206P
Supplier	Prime Polymer Co. Ltd.
Melt density	0.79516 g/cm^3^
Solid density	0.92219 g/cm^3^
Specific Heat	3095 J/kg °C
Thermal conductivity	0.25 W/m °C
Maximum shear stress	0.1 MPa
Mould temperature	40–60 °C
Melt temperature	160–180 °C

**Table 2:** Recommended setting of input parameters (Reproduced from [33], with the permission of AIP publishing).

Filling Time (s)	5.5
Mould Temperature (°C)	50
Melt Temperature (°C)	170
V/P switch-over (mm)	22.50
Packing time (s)	18.03
Packing pressure (MPa)	15.15
Cooling Time (s)	22.32
Shear Rate (1/s)	1437.0

**Table 3:** Input parameters of the thick plate part.

Factor	Levels	
	Low	High
Mould Temperature (°C)	40	60
Melt Temperature (°C)	160	180
Packing pressure (MPa)	12.20	18.56
Cooling time (s)	19.42	26.54

**Table 4:** Parameter values and responses.

Run	Setting Parameters				Responses		
	Mould Temperature (°C)	Melt Temperature (°C)	Packing Pressure (MPa)	Cooling Time (s)	Shrinkage in *x* Direction (%)	Shrinkage in *y* Direction (%)	Tensile Strength (MPa)
1	40	160	12.2	19.42	2.77	2.12	8.99
2	60	160	12.2	19.42	3.03	2.20	9.34
3	40	180	12.2	19.42	2.72	2.25	9.38
4	60	180	12.2	19.42	2.69	2.32	9.38
5	40	160	18.56	19.42	2.43	2.10	9.73
6	60	160	18.56	19.42	2.52	2.22	9.63
7	40	180	18.56	19.42	2.25	1.96	9.78
8	60	180	18.56	19.42	2.50	1.93	9.79
9	40	160	12.2	26.54	2.89	2.21	9.48
10	60	160	12.2	26.54	2.63	2.35	9.76
11	40	180	12.2	26.54	2.78	2.28	9.73
12	60	180	12.2	26.54	2.55	2.40	9.76
13	40	160	18.56	26.54	2.43	2.13	9.39
14	60	160	18.56	26.54	2.21	2.34	9.51
15	40	180	18.56	26.54	2.15	1.98	9.67
16	60	180	18.56	26.54	2.08	2.32	9.46
17	50	170	15.38	22.98	2.20	2.07	9.47
18	50	170	15.38	22.98	2.15	2.07	9.43
19	50	170	15.38	22.98	2.19	2.12	9.45
20	50	170	15.38	22.98	2.13	2.09	9.41
21	40	170	15.38	22.98	1.87	2.13	9.42
22	60	170	15.38	22.98	1.88	2.23	9.50
23	50	160	15.38	22.98	2.57	2.24	9.60
24	50	180	15.38	22.98	2.31	2.18	9.77
25	50	170	12.2	22.98	2.89	2.27	9.62
26	50	170	18.56	22.98	2.67	2.14	9.55
27	50	170	15.38	19.42	2.54	2.29	9.10
28	50	170	15.38	26.54	2.53	2.24	9.21
29	50	170	15.38	22.98	2.19	2.12	9.47
30	50	170	15.38	22.98	2.11	2.07	9.41

**Table 5:** Contribution factors in the *x* and *y* directions affecting the shrinkage.

	% Contribution of Shrinkage	
Parameters	*x* Direction	*y* Direction
A. Mould Temperature	14.60	18.71
B. Melt Temperature	14.60	16.65
C. Packing pressure	47.31	36.47
D. Cooling Time	0.58	1.50

**Table 6:** Simulation results.

Tools/Parameters		Mould Temperature (°C)	Melt Temperature (°C)	Packing Pressure (MPa)	Cooling Time (s)	Shrinkage Percentages (%)
Simulation (recommended)		50	170	15.2	22.32	(*x*) = 1.95% (*y*) = 1.85%
RSM	(*x*-direction)	40	160	18.6	26.54	1.78%
	(*y*-direction)	40	160	18.6	26.14	1.69%
PSO	(*x*-direction)	40	161	18.6	25.35	1.78%
	(*y*-direction)	53	160	18.5	22.33	1.67%

**Table 7:** The most contributing processing parameters.

Specimens		R0	R30	R40	R50
**Most significant parameter**	*x* direction	Packing pressure	Packing pressure	Packing pressure	Packing pressure
	*y* direction	Packing pressure	Mould temperature	Mould Temperature	Mould Temperature
	Tensile strength	Packing pressure	Packing pressure	Mould Temperature	Mould temperature

**Table 8:** Optimal shrinkage and tensile strength values.

Responses		Specimens	Mould Temperature (°C)	Melt Temperature (°C)	Packing Pressure (MPa)	Cooling Time (s)	Optimal Values
RSM	Shrinkage in *x* direction	R0	59.20	175.41	15.85	22.21	1.85%
		R30	42.41	160	18.56	19.42	2.45%
		R40	41.42	160	18.51	19.42	2.65%
		R50	46.53	170.42	16.49	19.48	2.80%
	Shrinkage in *y* direction	R0	40.35	179.77	18.48	22.65	1.92%
		R30	48.03	178.92	12.82	24.43	2.01%
		R40	49.36	180	12.72	25.23	2.05%
		R50	46.92	177.15	13.96	25.27	2.08%
	Tensile strength	R0	49.95	179.96	12.46	24.08	9.86 MPa
		R30	57.36	167.43	18.5	23.84	9.81 MPa
		R40	51.67	177.31	14.39	19.49	9.66 MPa
		R50	59.56	167.79	16.18	23.96	9.57 MPa
PSO	Shrinkage in *x* direction	R0	60.00	174.16	16.08	23.62	1.71%
		R30	40.48	160.25	18.25	19.72	2.56%
		R40	41.46	160.35	18.55	19.66	2.75%
		R50	40.09	170.58	17.88	19.48	2.75%
	Shrinkage in *y* direction	R0	40.09	179.91	18.41	22.67	1.92%
		R30	48.7	179.88	18.54	24.26	1.87%
		R40	48.77	179.62	12.28	26.50	2.01%
		R50	47	179.96	12.33	26.48	1.97%
	Tensile strength	R0	52.46	179.8	18.52	22.01	9.97 MPa
		R30	59.92	165.05	18.51	25.34	9.71 MPa
		R40	49.95	179.88	12.78	19.43	9.71 MPa
		R50	59.99	167.92	12.38	26.47	9.62 MPa

**Table 9:** Validation test for shrinkage in the *x* direction.

Parameter/Response	R0		R30		R40		R50	
	RSM	PSO	RSM	PSO	RSM	PSO	RSM	PSO
Mould temperature (°C)	59.20	60.00	42.41	40.48	41.42	41.46	46.53	40.09
Melttemperature (°C)	175.41	174.16	160.00	160.25	160.00	160.35	170.42	170.58
Packing pressure (MPa)	15.85	16.08	18.56	18.25	18.51	18.55	16.49	17.88
Cooling time (s)	22.21	23.62	19.42	19.72	19.42	19.66	19.48	19.48
Shrinkage, % (by optimisation method)	1.85	1.71	2.45	2.56	2.65	2.75	2.80	2.75
Shrinkage, % (validation test)	1.95	1.86	2.49	2.68	2.67	2.79	3.02	2.90
Error (%)	5.13	8.07	1.61	4.48	0.75	1.43	7.28	5.17

Remarks: R0: 100:0 recycled ratio blend; R30: 70:30 recycled ratio blend; R40: 60:40 recycled ratio blend; R50: 50:50 recycled blend.

**Table 10:** Validation test for shrinkage in the *y* direction.

Parameter/Response	R0		R30		R40		R50	
	RSM	PSO	RSM	PSO	RSM	PSO	RSM	PSO
Mould temperature (°C)	40.35	40.09	48.03	48.70	49.36	48.77	46.92	47.00
Melt temperature (°C)	179.77	179.91	178.92	179.88	180.00	179.62	177.15	179.96
Packing pressure (MPa)	18.48	18.41	12.82	18.54	12.72	12.28	13.96	12.33
Cooling time (s)	22.65	22.67	24.43	24.26	25.23	26.50	25.27	26.48
Shrinkage (by optimisation method) (%)	1.92	1.92	2.01	1.87	2.05	2.01	2.08	1.97
Shrinkage (validation test) (%)	2.08	2.01	2.08	2.05	2.16	2.11	2.19	2.10
Error (%)	7.69	4.48	3.37	8.78	5.09	4.74	5.02	6.19

Remarks: R0: 100:0 recycled ratio blend; R30: 70:30 recycled ratio blend; R40: 60:40 recycled ratio blend; R50: 50:50 recycled blend.

**Table 11:** Validation test for tensile strength of part.

Parameter/Response	R0		R30		R40		R50	
	RSM	PSO	RSM	PSO	RSM	PSO	RSM	PSO
Mould temperature (°C)	49.95	52.46	57.36	59.92	51.67	49.95	59.56	59.56
Melt temperature (°C)	179.96	179.80	167.43	165.05	177.31	179.88	167.79	167.79
Packing pressure (MPa)	12.46	18.52	18.50	18.51	14.39	12.78	16.18	16.18
Cooling time (s)	24.08	22.01	23.84	25.34	19.49	19.43	23.96	23.96
Tensile strength (by optimisation method) (MPa)	9.86	9.97	9.81	9.71	9.66	9.71	9.57	9.62
Tensile strength (validation test) (Mpa)	9.73	9.77	9.69	9.58	9.64	9.54	9.45	9.56
Error (%)	1.34	2.05	1.24	1.36	0.21	1.75	1.27	0.62

Remarks: R0: 100:0 recycled ratio blend; R30: 70:30 recycled ratio blend; R40: 60:40 recycled ratio blend; R50: 50:50 recycled blend.

## Data Availability

Data sharing not applicable.
